# Supplementary material for: Nutrient sensitive protein O-GlcNAcylation modulates the transcriptome through epigenetic mechanisms during embryonic neurogenesis
Source: Life Sci Alliance. 2022 Apr 25;5(8):e202201385. doi: 10.26508/lsa.202201385 (PMC9039347; doi:10.26508/lsa.202201385)
Supplement: Supplementary file 5 [file LSA-2022-01385_TableS1.docx]

**Supplementary table 1.** Blood glucose levels were analyzed in control and animals treated with streptozotocin.

| **Control group** | **Glucose level (mg/dL)** | **Hyperglycemia group** | **Glucose level (mg/dL)** |
| --- | --- | --- | --- |
| E14.5_R1 | 129 | E14.5_R1 | 597 |
| E14.5_R2 | 109 | E14.5_R2 | 427 |
| E14.5_R3 | 95 | E14.5_R3 | 481 |
| E14.5_R4 | 116 | E14.5_R4 | 461 |
| E16.5_R1 | 84 | E16.5_R1 | 600 |
| E16.5_R2 | 114 | E16.5_R2 | 440 |
| E16.5_R3 | 95 | E16.5_R3 | 511 |
| E18.5_R1 | 76 | E18.5_R1 | 479 |
| E18.5_R2 | 113 | E18.5_R2 | 528 |
| E18.5_R3 | 121 | E18.5_R3 | 455 |
| E18.5_R4 | 105 |  |  |
| E18.5_R5 | 118 |  |  |
| E18.5_R6 | 104 |  |  |
