# Supplementary material for: Nutrient sensitive protein O-GlcNAcylation modulates the transcriptome through epigenetic mechanisms during embryonic neurogenesis
Source: Life Sci Alliance. 2022 Apr 25;5(8):e202201385. doi: 10.26508/lsa.202201385 (PMC9039347; doi:10.26508/lsa.202201385)
Supplement: Supplementary file 6 [file LSA-2022-01385_TableS2.docx]

**Supplementary table 2:** A list of antibodies used in this study.

| **Antibody** | **Source** | **Catalog No.** |  |
| --- | --- | --- | --- |
| Anti-phospho-Ser/Thr-Pro MPM-2 Antibody | Millipore | 05-368 |  |
| EzH2 polyclonal antibody | Protintech | 21800-1-AP |  |
| Monoclonal Anti-β-Tubulin Isotype III antibody produced in mouse | Sigma-Aldrich | T-5076 |  |
| Purified anti-PAX-6 | Biolegend | 901301 |  |
| Anti-Tbr1 Antibody | Millipore | AB2261 |  |
| GAPDH (14C10) Rabbit mAb | Cell Signaling Technology | 2118 |  |
| Anti O-Linked N-Acetylglucosamine antibody [RL2] | | Abcam | ab2739 |
| Anti-PCNA antibody | | Abcam | Ab18197 |
| β-Actin Antibody (C4) | | Santa Cruz Biotechnology | sc-47778 |
| Ubiquityl-Histone H2B (Lys120) (D11) Monoclonal Antibody | | Cell Signaling Technology | 5546 |
| Anti-Histone H2B antibody-ChIP grade | | Abcam | Ab1790 |
| Anti-Histone H2B (GlcNAc S112) antibody | | Abcam | Ab130951 |
| Anti-Ubiquityl-Histone H2B antibody, clone 56 | | Millipore | 05-1312 |
| Anti-Histone H3 antibody | | Abcam | ab1791 |
| H3K4me3 Antibody-ChIP-seq Grade | | Diagenode | C15200152 |
| Histone H3K27ac (Acetyl H3K27) Polyclonal antibody | | Epigentek | A-4708 |
| Histone H3K27me3 (H3K27 Trimethyl) Polyclonal antibody | | Epigentek | A-4039 |
| Go-ChIP-Grade™ Purified anti-RNA Polymerase II RPB1 Antibody (8WG16) | | BioLegend | 664912 |
| Anti-RNA polymerase II subunit B1(phosphor CTD Ser-5) clone 3E8 | | Millipore | 04-1572 |
| Anti-MLL2 (C-Term) Antibody | | Millipore | ABE206 |
| MLL1 Antibody | | Bethyl Laboratories | A300-086A |
| H3K4me2 Monoclonal Antibody | | Diagenode | C15200151 |
| Phospho-EZH2 (Thr311) Antibody | | Cell Signaling Technology | 27888 |
| Phospho-EZH2 (Thr487) Polyclonal Antibody | | Invitrogen | PA5-105660 |
| LSD1 Antibody | | Cell Signaling Technology | 2139 |
| Purified polyclonal Rabbit IgG | | BioRad | PRABP01 |
| Peroxidase AffiniPure Goat Anti-Mouse IgG, Fcγ Subclass 2a Specific | | Jackson Immuno Research | 115-035-207 |
| Peroxidase AffiniPure Goat Anti-Mouse IgG (H+L) | | Jackson Immuno Research | 115-035-166 |
| Peroxidase AffiniPure Goat Anti-Rabbit IgG (H+L) | | Jackson Immuno Research | 111-035-144 |
| Peroxidase AffiniPure Rabbit Anti-Chicken IgY (IgG) (H+L) | | Jackson Immuno Research | 303-035-003 |
