# Supplementary material for: Nutrient sensitive protein O-GlcNAcylation modulates the transcriptome through epigenetic mechanisms during embryonic neurogenesis
Source: Life Sci Alliance. 2022 Apr 25;5(8):e202201385. doi: 10.26508/lsa.202201385 (PMC9039347; doi:10.26508/lsa.202201385)
Supplement: Supplementary file 7 [file LSA-2022-01385_TableS3.docx]

**Supplementary table 3.** A list of human (h) and rat (r) specific oligonucleotide sequences used for ChIP-qPCR.

| **Primer Name** | **5`-3` sequence** |  |
| --- | --- | --- |
| hTBR1-P1-Forward | TCATGAGGGCAAATGGCAGT |  |
| hTBR1-P1-Reverse | AGCTGTTGAATCGTTGGGGT |  |
| hTBR1-P2-Forward | GGCAGATAAAAAGCCTCTGCC |  |
| hTBR1-P2-Reverse | CGAGGTGTAGGGAGGAAGTCT |  |
| hEOMES-P1-Forward | CGTTAGGAGCCGGGTACACA |  |
| hEOMES-P1-Reverse | AGCGGTACTACCTCCAGTCC |  |
| hNEUROD1-P1-Forward | ACGTGACCTGCCCATTTGTA |  |
| hNEUROD1-P1-Reverse | GTCCGCGGAGTCTCTAACTG |  |
| hNEUROD1-P2-Forward | TAGATCAGAGCGAGTGGCCT |  |
| hNEUROD1-P2-Reverse | CGAAAGGAGCGAGGACTCTT |  |
| hFOXG1-P1-Forward | CCCTTATCCAAAGCTGCGCTA |  |
| hFOXG1-P1-Reverse | GCGAGGCACTACTTTTCCGA |  |
| hFOXG1-P2-Forward | GCGGTAGGAAAGGTTAAACCAA |  |
| hFOXG1-P2-Reverse | AGCGATCGAGGCGGCTAT |  |
| hPAX3-P1-Forward | CCGGGACAATTTCGAGACAAC |  |
| hPAX3-P1-Reverse | TTTTGGAAATGCCGCGGTG |  |
| hPAX3-P2-Forward | TTAAGAGAACAGGCGGGCAG |  |
| hPAX3-P2-Reverse | GTCCCAGAGGATTCAGCGAG |  |
| hTBX3-P1-Forward | | AGGATGCGGCCATATCTCAG |
| hTBX3-P1-Reverse | | GATACACCGGGGGTGTTTGG |
| hTBX3-P2-Forward | | GTCCTTGTGCCTTGCGTTTT |
| hTBX3-P2-Reverse | | TCTATTTCGAGCTCAGCGGC |
| hHOPX-P1-Forward | | TTTCGCAGACAGACCAGAGG |
| hHOPX-P1-Reverse | | CCCGGGATACACGCTTTCTC |
| hHOPX-P2-Forward | | TCTCCCTTTCACCTCTACCGT |
| hHOPX-P2-Reverse | | GGTCTGCCTTTTATGCAGAGGA |
| hNGN1-S5-Forward | | AAGAGTGGACCCTTCTGCTTG |
| hNGN1-S5-Reverse | | ACTCCACATCAGCTGGCTCT |
| hNGN2-S5-Forward | | AGAAACAGAGGCGCGATCTT |
| hNGN2-S5-Reverse | | GACCTTGGTTAGACTGCCT |
| hHES5-TSS-Forward | | CTATATAGGCGCGGGCGG |
| hHES5-TSS-Reverse | | TCACAGGCAATTTAGCGTGC |
| hNR2E1-TSS-Forward | | CGGGAGGTGTAACCTCTGAC |
| hNR2E1-TSS-Reverse | | AGCGATGATCCCCGATCTCT |
| hPAX3-TSS-Forward | | TTCGTGTTAGTGAACCGCCC |
| hPAX3-TSS-Reverse | | TTCAAGAGCTGAGCCGCTA |
| hFOXG1-TSS-Forward | | TGATCAATGTGCTCCCCACT |
| hFOXG1-TSS-Reverse | | AATGGGGAAATTGCGAATCGG |
| hFOXG1-S5-Forward | | CTGTGAGATCCACGTTCCCA |
| hFOXG1-S5-Reverse | | CGGGCCTCATTCCTGAAATA |
| hTBR1-TSS-Forward | | AGGCAGCTTTGGGCAGATAA |
| hTBR1-TSS-Reverse | | CCGAGGTGTAGGGAGGAAGT |
| hNEUROD1-TSS-Forward | | TCACTACGTCAGTCCCCACC |
| hNEUROD1-TSS-Reverse | | CTCCCACATAAGAGAGCGCA |
| hFOXP2-TSS-Forward | | ACAGGAACCAGGGCAACAAT |
| hFOXP2-TSS-Reverse | | TGCGTTAAAGCGCTGATTGG |
| hEOMES-TSS-Forward | | CCAAAAGCTTAGCGCGAGTT |
| hEOMES-TSS-Reverse | | AACTCCCTGGCCTTCAACG |
| hFEZF1-TSS-Forward | | ACCAATGACTCGGGGACAATC |
| hFEZF1-TSS-Reverse | | CTGTTGACTGTTGGAAGCGA |

| **Primer Name** | **5`-3` sequence** |
| --- | --- |
| rTBR1-P-Forward2 | AGTTTTACAGGGCGAGAAGAGG |
| rTBR1-P-Reverse2 | GGTGGAGGAGGGAATTCGATG |
| TrTBR1-S5-Forward2 | GGTCATCACCAACGGAGCTT |
| rTBR1-S5-Reverse2 | TTGGTAGGAGTGGCCGTACT |
| rFOXG1-P-Forward2 | CCCGATTGGTCGACGGCTA |
| rFOXG1-P-Reverse2 | GAGCTACAGGCGCACACTA |
| rFOXG1-S5-Forward2 | GTTCAGCTACAACGCGCTCA |
| rFOXG1-S5-Reverse2 | CGCGGTAGTAAGGGAAGTTCT |
| rTBX3-S5-Forward1 | TTCAAATTGAGGAACGGGTGG |
| rTBX3-S5-Reverse1 | TTTCTCTCTCTTCCCCAGGGTT |
| rNGN2-S5-Forward1 | CAACCGCATGCACAACCTG |
| rNGN2-S5-Reverse1 | GTGAGCGCCCAGATGTAATTG |
| rNEUROD1-P-Forward1 | CTAACTGGCGACAGATGGGC |
| rNEUROD1-P-Reverse1 | CCATATGGTCTTCCCGGTCC |
| rNEUROD1-S5-Forward1 | AACAGGAAGTGGAAACATGACCA |
| rNEUROD1-S5-Reverse1 | AGAACTGAGGCACTCGTCTGTC |
| rEOMES-P-Forward1 | TGACACTTTGGGTCGCTCTC |
| rEOMES-P-Reverse1 | GGGTCTAGTTTGGCAGGTCC |
| rEOMES-S5-Forward1 | CCCAGAATCTCCCAACACCG |
| rEOMES-S5-Reverse1 | GCGTGTTGTTGTTGTTAGCG |
| rFOXP2-P-Forward1 | CGGGGCAGAACAAACAAGTTC |
| rFOXP2-P-Reverse1 | AATCTCGGGCCTTAGAGTCG |
| rFOXP2-S5-Forward1 | ACCCGCAGTTCATCCGTCAG |
| rFOXP2-S5-Reverse1 | CGAACAACACACCCACGATC |

P = promoter; TSS = transcription start site; S5 = 5’ end of gene
